# Assessing partial errors via analog gaming keyboards in response conflict tasks: A proof-of-concept study with the concealed information test

**DOI:** 10.3758/s13428-022-02039-4

**Published:** 2023-01-03

**Authors:** Dave Koller, Franziska Hofer, Bruno Verschuere

**Affiliations:** 1 Department of Psychology, University of Zurich, Binzmühlestrasse 14 / Box 22, 8050 Zurich, Switzerland; 2 Department of Clinical Psychology, University of Amsterdam, Amsterdam, The Netherlands; 3 Zurich State Police, Airport Division, Research and Development, Zurich, Switzerland; 4 HF Partners, Zurich, Switzerland

**Keywords:** Memory detection, Concealed Information Test, CIT, Deception, Sternberg task, Response tendency, Analog keyboard

## Abstract

The response time-based Concealed Information Test (RT-CIT) is an established memory detection paradigm. Slower RTs to critical information (called ‘probes’) compared to control items (called ‘irrelevants’) reveal recognition. Different lines of research indicate that response conflict is a strong contributor to this RT difference. Previous studies used electromyography (EMG) to measure response conflict, but this requires special equipment and trained examiners. The aim of this study was to explore if response conflict can also be measured with an analog gaming keyboard that is sensitive to minimal finger movements. In a preregistered study, participants completed an autobiographical RT-CIT (*n* = 35) as well as a cued recognition task (modified Sternberg task; *n* = 33) for validation purposes. Partial errors, partial button presses of the incorrect response key, were more frequent in trials with response conflict than in trials without conflict. Partial errors were rare (CIT: 2.9%; Sternberg: 1.7% of conflict trials), suggesting analogue keyboards have lower sensitivity than EMG. This is the first evidence that analog keyboards can measure partial errors. Although likely less sensitive than EMG measures, potential benefits of analog keyboards include their accessibility, their compatibility with all tasks that use a standard keyboard, that no physical contact with the participant is needed, and ease of data collection (e.g., allowing for group testing).

## Introduction

The Concealed Information Test (CIT) aims to detect if someone has specific knowledge that they cannot or do not want to reveal (Lykken, 1959). Examinees are presented with several, equally plausible pieces of information (e.g., examinee’s own name ALEX amongst a series of irrelevant names like FRANK, DAVID, and MARK) and they are asked to indicate whether they recognize the information. The concealed item typically elicits a distinct behavioral (Seymour et al., 2000), physiological (Lykken, 1959), and neurophysiological response (Langleben et al., 2002; Rosenfeld et al., 1988, 2008) compared to the irrelevant items, that can be used to infer recognition of the presented information (for a review, see Verschuere and Meijer, 2014).

Because of its simplicity and its validity, there is a renewed interest in behavioral responding, response times (RTs), specifically (see e.g., the machine leaning mega-analysis by Lukacs and Steyrl (2022); for a meta-analytic review see Suchotzki et al., 2018). The response time-based CIT (RT-CIT) effect – the slower responding to concealed information than to control items – has been linked to response conflict and response inhibition (Seymour & Schumacher, 2009; Schumacher et al., 2010; Suchotzki et al., 2015). Apart from the concealed items (also called *probe* items) and the control items (also called *irrelevant* items), the RT-CIT additionally has so-called *target* items. Targets are items to which examinees are instructed to respond differently than to all other items (i.e., press YES when you recognize the target; Farwell & Donchin, 1991). Targets are typically learned before the test and are therefore familiar to the participant. Because familiarity is a valid cue that is in line with recollection for irrelevant and target items (which make up five out of six trials) and because the RT-CIT is a speeded paradigm, participants might strongly rely on the fast familiarity-based responding (Ratcliff & McKoon, 2008; Yonelinas, 2002). For probes, however, the familiarity-based response (YES, because it is familiar) contradicts the recollection-based response (NO, because recognition should be concealed) which is expected to lead to response conflict and therefore slower RTs.

Different lines of research have been used to test the presumed role of response conflict in the RT-CIT. One line of research aimed to manipulate response conflict in the RT-CIT experimentally. Lukács et al. (2017) added familiarity related “filler” items (e.g., the word “FAMILIAR” or “UNFAMILIAR”) to the RT-CIT which needed to be classified as familiar or unfamiliar. They argued that these filler items could increase the reliance on familiarity and therefore should increase response conflict. While they found larger probe-irrelevant RT differences in the filler condition (replicated by Olson et al., 2020), they note that this could also be due to deeper semantic encoding or disruption of a target focused response strategy (also see Koller et al., 2021). A more direct approach that did not modify the RT-CIT paradigm, and also succeeded in increasing the RT difference, is using personally familiar instead of learned targets (Suchotzki et al., 2018). The reasoning behind this manipulation is similar as for the fillers: Familiarity-based responding becomes a more viable strategy to do the CIT, since targets and irrelevants can be classified correctly and quickly based on familiarity alone. For probes, however, familiarity is an invalid cue and familiarity-based responding needs to be inhibited. Increasing target familiarity probably also increased target saliency and therefore the response conflict due to overlap in the saliency dimension between targets and probes. Since we are interested in response conflict in general, this is not problematic, but the manipulation also introduced differences in task difficulty (of the RT-CIT with versus without fillers) as a possible confound (see also Lukács & Ansorge, 2021). The familiar target condition might be easier because targets did not need to be learned and retained.

Another line of research investigated the mechanisms involved in the CIT using neurophysiological measures linked to response conflict detection and resolution. fMRI studies showed increased activation in the ventral fronto-parietal network for probes compared to irrelevants (for a meta-analysis, see Gamer, 2011). This network is connected to multiple potentially important mechanisms for the CIT like response inhibition (Zhang et al., 2017), but also to attention (Strange et al., 2000), and memory (Nyberg et al., 2003) which complicates isolated inferences about one of those mechanisms (i.e., the reverse inference problem). Furthermore, the insights from fMRI-based CIT studies – that typically have a slower pace and no targets – might not be directly transferable to the RT-CIT. Turning to the EEG, the N200 has been linked to conflict monitoring (Huster et al., 2013), and a recent meta-analysis found deception to be associated with a more negative N200 than truth telling (Sai et al., 2022). However, Huster et al. (2013) also acknowledged that the precise process leading to an N200 remains to be elucidated. Also, attempts to link measures of executive control to probe-irrelevant differences in RTs did not provide evidence for a connection (Suchotzki et al., 2015; Visu-Petra et al., 2012, 2014).

A more direct approach to measure response conflict in the RT-CIT used electromyography (Seymour & Schumacher, 2009; for a related approach see Hadar et al., 2012). Electrodes were placed on the triceps brachii of each arm to measure muscle activity. Participants held two cylinders with electric switches and responded ‘old’ to targets and ‘new’ to probes and irrelevants by exerting a “moderate downward force” (Seymour & Schumacher, 2009, p. 76) to those cylinders. This study found that probes elicited subthreshold muscle activity in the arm indicating recognition more frequently than irrelevant items. These so-called partial errors were used as evidence for response conflict in other conflict tasks before (e.g., Eriksen, Coles, Morris, & O’Hara, 1985; Coles, Gratton, Bashore, Eriksen, & Donchin, 1985) and are considered small corrected errors (e.g., Allain et al., 2009). By measuring response-related muscle activity, electromyography can provide strong evidence for response tendencies and response conflict, but it comes with its drawbacks. It requires specialized equipment, trained personnel to place the electrodes correctly, and often requires adaptations of well-established experimental tasks that typically use a keyboard.

Could partial errors also be assessed with a commercial analog gaming keyboard which not only registers if a key is pressed or not but how far a key is pressed at any given time? Such would provide us with a relatively simple tool to detect response conflict in individual trials for a wide array of RT-tasks without the need to modify the experimental paradigm. Sure enough, researchers have used custom-made devices for this purpose (see e.g., Li, Latash, Newell, & Zatsiorsky, 1998), but building and maintaining such devices requires engineering skills, which not all researchers have (access to). For the RT-CIT, partial button presses could also increase classification performance or help detect countermeasures. Just like the partial errors picked up by the electromyogram, we expect that response conflict leads to partial errors in the form of partial button presses (Seymour & Schumacher, 2009; the precise definition is provided in the Method section). We also manipulated the amount of response conflict in the CIT by using familiar versus unfamiliar targets (Suchotzki et al., 2018). From this, we derived the following four main hypotheses. The first two hypotheses pertain to the benchmark probe-irrelevant difference in RTs and the replication of Suchotzki et al. (2018) on the effect of familiar targets on RTs: (1) Probes show larger RTs than irrelevant items and (2) the probe-irrelevant difference in RTs is larger in the high familiarity condition (i.e., familiar targets) compared to the low familiarity condition (i.e., learned targets). Since we expect partial button presses to measure response conflict, we predicted the same effects for partial button presses: (3) partial button presses occur more frequently for probes than for irrelevant items and (4) we expect a larger probe-irrelevant difference in the frequency of partial button presses in the high familiarity condition compared to the low familiarity condition.

While our focus is on the RT-CIT, partial button presses should also occur in other, non-deceptive, conflict tasks. To ensure that partial button presses are not unique to the RT-CIT and that potential differences between the familiarity conditions are not due to task difficulty, we employed the modified Sternberg task (Oberauer, 2001), a cued recognition task, as a secondary response conflict task. Conflict was manipulated by the proportion of trials for which familiarity is a valid cue (match and new trials; see Method section) compared to intrusion trials for which familiarity induces response conflict. For this additional task, we had the following hypotheses: (5) ’An ‘intrusion cost’ is expected, i.e., (RTintrusions minus Rtnew) > 0^1^. (6) Intrusion costs in the high-conflict condition are larger than in the low-conflict condition. Concerning partial button presses, we expected that (7) partial button presses occur more frequently in intrusion trials compared to new trials and that (8) the difference in the frequency of partial button presses between intrusions and new trials as well as between intrusions and matches is larger in the high-conflict condition than in the low-conflict condition.

## Method

The experiment was approved by the ethics committee of the Faculty of Social and Behavioural Sciences of the University of Amsterdam (approval number: 2020-CP-12001). Preregistration, material, data, and scripts can be found on https://osf.io/x8ecn/. The two tasks were programmed with MATLAB version 9.4.0 (The MathWorks, 2018) with the Psychtoolbox extension version 3.0.14 (Brainard, 1997).

### Deviations from preregistration

One Swiss participant was tested at the University of Amsterdam, although only German and Dutch participants were preregistered as eligible. However, this criterion was based on the demographics of students at the University of Amsterdam and not on the study design. Because the inclusion of this participant does not diminish the validity of this study in any way, we decided to not exclude this participant.

### Partial button presses

We used the Wooting Two Lekker edition keyboard to measure partial button presses (see https://wooting.io/wooting_two_lekker). This gaming keyboard uses hall effect switches to translate the position of any key into an analog value ranging from 0 to 1. We installed Wootility Lekker (Version 4.1.2. beta). To read out the analog values, we used the Wooting Analog SDK (version 0.2.0). Both can be found on OSF (https://osf.io/x8ecn/).

Keys that are not pressed down have an analog value of 0, fully pressed keys have an analog value of 1. However, our pre-testing showed that if a key is pressed at an angle, the value might not quite reach 1. Therefore, we decided to set the threshold of when we consider a key to be fully pressed to analog values > .95. The analog values were retrieved at a rate of 1000 Hz. To reduce the size of the data files, we only recorded the analog values and the corresponding timestamp when the analog value changed since the last retrieval. We speak of a partial button press if both response keys showed analog values > 0 before the response threshold (analog value > .95) was reached.

### Participants

Participants were eligible to enroll if they were at least 18 years old and if they have moved at least once in the past 5 years. Data were collected simultaneously at the University of Zurich and the University of Amsterdam. Participating at the University of Zurich required proficiency in German and one of the following nationalities: Swiss, German, or Austrian. Participants at the University of Amsterdam were required to be proficient in English and either Dutch or German. Completion of this study took participants about 75 min and was reimbursed according to the standard rates of the respective universities (19 CHF at the University of Zurich, 12.50 EUR at the University of Amsterdam). Participants were recruited via a participant mailing list and via the research study platform of the University of Amsterdam.

Following the preregistered recruitment procedure, we concluded data collection based on our time deadline. A total of 43 participants were recruited but two participants were excluded prior to data analysis due to illegibility or technical errors. Of the 41 participants that entered the data analysis, five (12%) were excluded based on the preregistered language proficiency criteria (LexTALE score > 70; Lemhöfer & Broersma, 2012). One participant had to be excluded from the RT-CIT because the RT-CIT could not be constructed due to item familiarity (see below) resulting in a sample of *n* = 35 (M age = 25.89, SD = 5.14, range 18–38 years, 80% female) for the RT-CIT (*n* = 19 in the low familiarity condition, and *n* = 16 in the high familiarity conditions). Three participants had to be excluded from the modified Sternberg task due to poor task performance (less than 60% correct in at least one item category) resulting in a final sample *n* = 33 (M age = 25.33, SD = 4.59, range 18–36 years, 78.8% female) for the modified Sternberg task (*n* = 18 in the low-validity, and *n* = 15 high-validity conditions). Of the 36 participants, 26 (72.2%) participated at the University of Zurich (22 Swiss, three German, one Austrian) and ten at the University of Amsterdam (seven Dutch, two German, one Swiss; see deviations from preregistration).

### Procedure

The experimenter welcomed the participants and asked them to read and sign the informed consent. It was clearly stated that participation is voluntary and that participants can withdraw their consent at any time without giving reasons or disadvantages. They were further informed that data containing their personal information will be treated confidentially and that an anonymized version of the data will be made publicly accessible on a data repository. After providing consent, participants then completed the RT-CIT and the modified Sternberg task. The task order was balanced between participants (before exclusions). After the two response time tasks, participants completed the LexTALE language proficiency task (Lemhöfer & Broersma, 2012). Finally, participants were debriefed, reimbursed, and thanked for their participation.

### RT-CIT

Before the RT-CIT started, we asked participants for autobiographical information (name, surname, date of birth as well as the street and city they currently live in). We also asked them to provide their former address (street and city) as well as the name, surname, and date of birth of a good friend of the same sex. The information was entered by the participant but under supervision of the experimenter to ensure that the format is consistent with the other items used in the RT-CIT (e.g., no abbreviations).

Next, we presented participants with lists of seven items, one list per information category (i.e., seven names, seven surnames, etc.), and asked them to indicate up to two items that were of personal relevance to them by clicking on them. Erroneous clicks could be corrected by clicking on the same item again. The indicated items were removed from the item pool that we used to construct the upcoming RT-CIT. Participants were instructed to contact the experimenter if more than two items in a list were of personal relevance because in that case, the RT-CIT could not be constructed.

We then asked the participants to imagine that they want to flee a country, but the police and border control are looking for them. This is why they carry a fake ID with them. They get stopped by the border control at the airport and tested for their identity. Participants were instructed to hide their true identity and to pretend to be the person on the fake ID whose information (i.e., name, surname, date of birth, street, and city) was shown on the screen (for similar scenarios see e.g., Verschuere & Kleinberg, 2016). To do so, they should press YES when presented with any information of the fake ID (targets) and NO for all other information (irrelevant items and probes). We asked participants to learn the information of their fake identity and tested their memory using free recall. Only participants without errors in the free recall could proceed to the RT-CIT. Participants were redirected back to the learning phase if they made an error.

The RT-CIT consisted of the five information categories (name, surname, date of birth, street, and city), with six items per category (one probe, one target, four irrelevant items; within-subjects factor). The true autobiographical information was used as probes. The irrelevant items were randomly selected from a pre-selected pool of potential irrelevant/target items (see https://osf.io/x8ecn/). Target items were either all randomly selected from the item pool (low familiarity condition) or the friend’s information and the participant’s previous address were used as targets (high familiarity condition; between-subjects factor).^2^ On each trial, a single item was presented in the middle of the screen. Participants were instructed to answer the question “Is this you?” as quickly and accurately as possible by pressing either “i” or “e” on the keyboard. The NO response was mapped to the participant’s dominant hand. Participants should keep their index fingers on the response keys throughout the RT-CIT. The items were displayed until a response was given or the response deadline was reached. The response-stimulus interval varied randomly between 500 and 1000 ms. However, if participants were pressing a response key when the next trial was supposed to start, a message to fully release all keys was displayed. The next trial started between 500 and 1000 ms after the keys were released.

The RT-CIT started with three practice blocks of 30 trials each, in which every item was presented once. A red “X” (in case of an error) or a red “TOO SLOW” message displayed for 200 ms below the item provided feedback in the practice phase. The “TOO SLOW” message was shown if the response time was larger than 10 s in the first practice block, larger than 1.2 s in the second practice block, or larger than 0.8 s in the third practice block. Response deadlines for the three practice blocks for were 10 s, 1.5 s, and 1.5 s, respectively. Participants had to repeat the third practice phase if they had less than 50% correct for any item type (probe, target, irrelevants) or a mean response time larger than 800 ms. Participants could do the practice phase up to four times. After the third time, however, participants were instructed to get the experiment leader to ensure that the task was understood properly. If participants failed the fourth practice phase, the experiment was terminated. The test phase consisted of 20 blocks of 30 trials each, resulting in 600 test trials in total (100 probes, 100 targets, and 400 irrelevant items). Every item was presented once per block and the response deadline was set to 1.5 s. Participants could take a short self-paced break after ten blocks. The RT-CIT was followed by a free recall of target items to ensure that participants did not forget the targets during the test.

### Modified Sternberg task

**Fig. 1:**
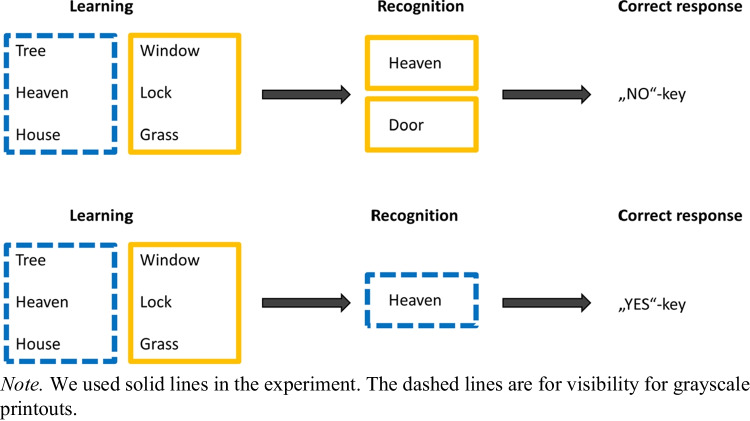
Illustration of the different trial types of the modified Sternberg tasks

The modified Sternberg task (Oberauer, 2001) is a cued recognition task (Fig. 1). The learning phase consisted of two lists of three nouns each that were presented side by side in colored rectangles (blue and yellow). The six items were presented simultaneously for 4.8 s followed by a blank screen of 800 ms. In the recognition test, one word was shown in either a blue or yellow rectangle. The participants’ task was to indicate as quickly and accurately as possible if the presented word was in the list of the cued color. There are three possible trial types (within-subjects factor: match, intrusion, new) depending on the word–color combinations. In a match trial, the word was in the list of the cued color. If the word was part of one list but is presented with the color of the other list, this is a so-called intrusion trial. Finally, if a word is presented that was not in either list, it is called a new trial. Match trials require a YES response while intrusion and new trials require a NO response. Like in the RT-CIT, “e” and “i” were the response keys and the NO response was mapped to the participant’s dominant hand. Participants were also instructed to keep their index fingers on the response keys throughout the task. The items were displayed until a response was given or the response deadline was reached. The response-stimulus interval varied randomly between 500 and 1000 ms. However, if participants were pressing a response key when the next trial was supposed to start, a message to fully release all keys was displayed. The next trial started between 500 and 1000 ms after the keys were released.

We manipulated the validity of familiarity as a cue to solve this task (low validity, high validity; between-subjects) by changing the proportion of new and intrusion trials. The low-validity condition consisted of 40% intrusion trials and 10% new trials, the high-validity condition used 15% intrusion trials and 35% new trials. The task consisted of 50% match trials in both conditions to ensure that there is no dominant response key. Consequently, purely familiarity-based responding would lead to 60% and 85% correct responses in the low- and high-validity condition, respectively.

The modified Sternberg task started with two practice blocks of ten trials each. A red “X” (in case of an error) or a red “TOO SLOW” message displayed for 500 ms below the item provided feedback in the practice phase. The “TOO SLOW” message was shown if the response time was larger than 6 s in the first practice block or larger than 1.5 s in the second practice block. Response deadlines for were 6 s and 2.5 s, respectively. The test phase consisted of 120 trials with a response deadline of 2.5 s. Participants could take a short self-paced break after 40 and 80 trials. Cue color and word position within the list for match and intrusion trials was balanced across test trials. No word was presented more than once.

### LexTALE

We used the MATLAB (The Math Works, 2018) based LexTALE versions provided on LexTALE’s website (www.lextale.com). The language tested by the LexTALE corresponded to the language of the RT-CIT and the modified Sternberg task (i.e., German for participants at the University of Zurich; English for participants at the University of Amsterdam). In this test, participants were presented with 60 strings of letters – 40 real words (e.g., scornful, ablaze), 20 pseudowords (e.g., mensible, pulsh) and their task was to indicate whether this string is a word of the tested language or not. If they recognized a word but did not know its meaning, they should still indicate “yes”. However, if they are unsure, they should indicate “no”. The LexTALE score is calculated as *% correct*_*av*_ = ((2.5*number of words correct) + (5*number of nonwords correct))/2. This score highly correlates with other language proficiency measures such as the Quick Placement Test (2001) (*r* = .63) and translational scores (*r* = .75; Lemhöfer & Broersma, 2012). For more detailed information about the LexTALE, see Lemhöfer and Broersma (2012).

After participant exclusions due to low scores in the LexTALE (*% correct*_*av*_ ≤ 70), participants had a mean score of *M % correct*_*av*_ = 85.3 (SD = 6.61; range 71.25–96.25). This corresponds to a high level of language proficiency (cf. Frank et al., 2019; Lemhöfer & Broersma, 2012).

## Analyses and results

Analyses were conducted in R (version 4.0.3; R Core Team, 2020) with the BayesFactor (Morey & Rouder, 2018) and brms (Bürkner, 2017) package.

### RT-CIT

#### Preregistered analyses

Following Koller et al., (2021), we excluded target trials, trials with response times smaller than 200 ms or larger than 1500 ms, and trials with response errors. We also excluded trials that start with a partially pressed key (analog value > 0 in the first 5 ms of a trial) to avoid accidental key presses. In total, 1.82% of probe and irrelevant trials were excluded. We then calculated average RT (*M* RT) for each participant in each condition.

**Fig. 2:**
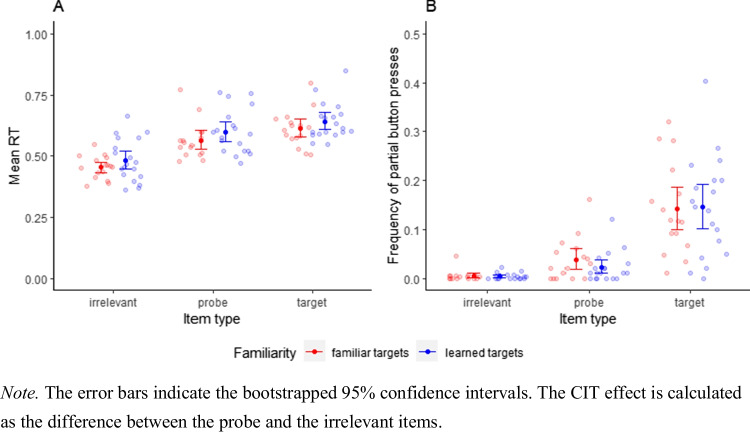
Participant mean RTs and frequency of partial button presses by item type in the RT-CIT

RTs. To test for the CIT effect in RTs (Hypothesis 1) and for the effect of target familiarity on the CIT effect in RTs (Hypothesis 2), we conducted a two (item type: probe vs. irrelevant; within-subjects) by two (target familiarity: learned targets vs. familiar targets; between-subjects) Bayesian mixed effects ANOVA with JZS priors (Cauchy priors with scale = .5) on the participant mean RTs (Fig. 2A). Comparing the main effects model M_Main_, the model with both main effects, to the model with only the main effect of familiarity (M_Fam_) showed that the data is much more likely under M_Main_ (BF_Main,Fam_ = 2.0*10^9^)^3^, providing strong evidence for the predicted probe-irrelevant difference in RTs (M RT_probe_ = 583 ms, SD = 75 ms versus M RT_irrelevant_ = 469 ms, SD = 72 ms). Comparison of the model with both main effects and the interaction (M_Full_) and M_Main_ showed anecdotal evidence *against* an interaction (BF_Full,Main_ = .33). In other words, the data is more likely under the model without the interaction than under the full model. Hypothesis 2, the increased probe-irrelevant difference in the familiar target condition (Suchotzki et al., 2018), was therefore not supported by the data. The results were robust to changes in the width of the Cauchy prior.

Partial button presses. We also predicted a CIT effect (Hypothesis 3), moderated by target familiarity (Hypothesis 4), for partial errors. Therefore, we tested these hypotheses in an analogous manner to the RT analyses. We conducted a two (item type: probe vs. irrelevant; within-subjects) by two (target familiarity: learned targets vs. familiar targets; between-subjects) Bayesian mixed effects ANOVA with JZS priors (Cauchy priors with scale = .5) on the frequency of partial button presses (Fig. 1B). The data were more likely under the main effects model than under the model with only a main effect of familiarity (BF_Main,Fam_ = 275)^4^, providing strong evidence for an effect of item type (Hypothesis 3). This means that the CIT effect was also apparent in the frequency of partial button presses (M Proportion partial presses_probes_ = 2.93% , SD = 3.73% versus M Proportion partial presses_irrelevants_ = .46%, SD = .88%). Comparing the full model to the main effects model showed anecdotal evidence against an interaction effect (BF_Full,Main_ = .59) and therefore against Hypothesis 4. The results did not qualitatively change when we used the arcsine transformed data and the results were robust to changes in the width of the Cauchy prior.

### Non-preregistered analyses

**Fig. 3:**
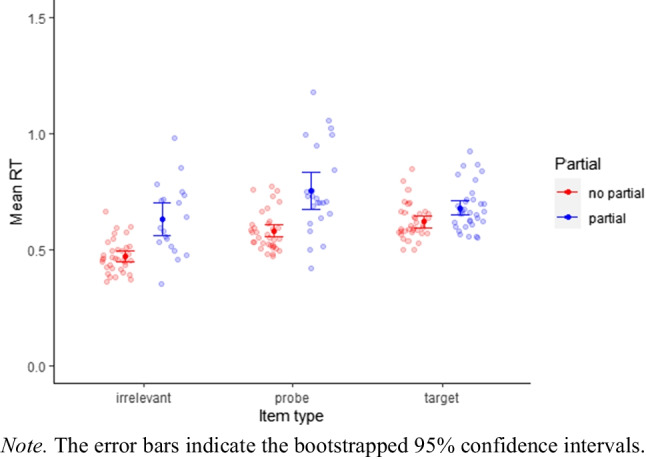
Comparison of participant mean RTs for trials with and without partial button presses

Because we did not find evidence for an effect of target familiarity in the preregistered analyses, we do not distinguish between the two groups in the exploratory analysis. We calculated the mean RTs for trials with and without partial button presses (see Fig. 3). Inspection of the figure suggests larger RTs for trials with partial button presses than for those without partial button presses. Also, it seems that difference is less pronounced for targets than for irrelevants and probes. However, since partial button presses are more frequent in target trials, aggregation gives more weight to partial button presses of irrelevant and probe trials than to target trials. (One person’s mean RT of probes with partial button presses might rely on very few trials while the mean RT of targets with partial button presses relies on more trials, but aggregation results in two data points with equal weight.) Therefore, we fitted an exponentially modified gaussian distribution model to the individual trial data using brms (Bürkner, 2017). The model included the main effects of item type and partial button press, their interaction, and random intercepts of participants and information category (e.g., name, surname, date of birth).

$${\displaystyle \begin{array}{l}\textrm{RT}\sim 1+\textrm{item}\ \textrm{type}\ast \textrm{partial}+\left(1|\textrm{participant}\right)+\left(1|\textrm{information}\right)\\ {}\textrm{sigma}\sim \textrm{item}\ \textrm{type}+\textrm{partial}\\ {}\textrm{beta}\sim \textrm{item}\ \textrm{type}+\textrm{partial}\end{array}}$$

We used the default priors, two chains, 10,000 iterations (including 1000 warm-up iterations) and set the adapt_delta parameter to .98. Rhat was 1.0 for all parameters, showing that convergence. A detailed description on the exponentially modified gaussian distribution model can be found on https://cran.r-project.org/web/packages/brms/vignettes/brms_families.html.

**Fig. 4:**
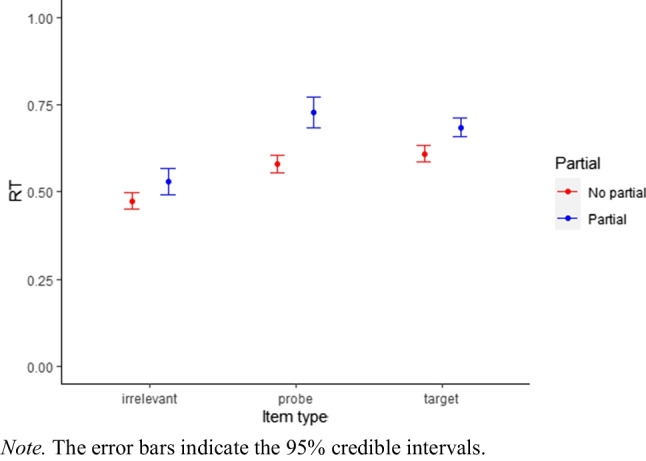
Conditional effects

The conditional effects (Fig. 4) showed larger RTs for trials with partial button presses compared to trials without partial button presses. The mean RT costs of partial errors varied with item type (irrelevant: M = 56 ms, probe: M = 149 ms, target: M = 77 ms), which could reflect the different stages at which the conflict occurs. For probes, we expected conflict when recollection provides the information that the correct response is ”no”, contrary to the familiarity based information. The expected conflict for targets is based on the predominant ”no”-response in the CIT (five out of six items require a ”no”-response) that conflicts with the familiarity based ”yes”-response. Therefore, conflict occurs before recollection information is available. For irrelevants, we did not expect any response conflict.

### Modified Sternberg task

#### Preregistered analyses

Trials with response times smaller than 200 ms or larger than 2500 ms, trials that start with a partially pressed key (analog value > 0 in the first 5 ms of a trial), and trials with response errors were excluded from the analysis; 617 out of 3960 trials (15.58%) were excluded (18.03% of match trials, 17.55% of intrusion trials, 7.21% of new trials). Out of the 617 excluded trials, 543 (88%) were excluded due to response error.

##### RTs

**Fig. 5:**
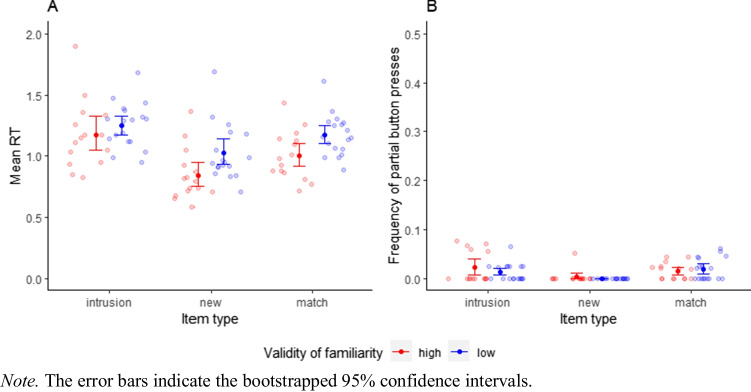
Participant mean RTs and frequency of partial button presses by item type in the modified Sternberg task

We conducted a two (item type: intrusion vs. new; within-subjects) by two (validity of familiarity: low vs. high; between-subjects) Bayesian mixed effects ANOVA with JZS priors (Cauchy priors with scale = .5) on the participant mean RTs (Fig. 5A). Comparing the main effects model (M_Main_) to the model with only the main effect of familiarity (M_Fam_) showed that the data is much more likely under M_Main_ (BF_Main,Fam_ = 1.7*10^9^)^5^. Therefore, we found strong evidence for intrusion costs in RTs (Hypothesis 5; M RT_intrusion_ = 1216 ms, SD = 231 ms versus M RT_new_ = 941 ms, SD = 237 ms). The comparison between the full model (M_Full_) and M_Main_ showed the data were about equally likely under the model with vs without the familiarity × item type interaction (BF_Full,Main_ = 1.46). With the BF_Full,Main_ being close to 1, the current data does not allow to reach a conclusion on the presence (or absence) of the interaction predicted by Hypothesis 6. The results were robust to changes in the width of the Cauchy prior.

##### Partial button presses

We conducted a three (item type: intrusion vs. new vs. match; within-subjects) by two (validity of familiarity: low vs. high; between-subjects) Bayesian mixed effects ANOVA with JZS priors (Cauchy priors with scale = .5) on the frequency of partial button presses (Fig. 5B). As predicted by Hypothesis 7, we found strong evidence for a main effect of item type (BF_Main,Familiarity_ = 127)^6^ but anecdotal evidence against an interaction effect (BF_Full,Main_ = .34), contrary to Hypothesis 8. Pairwise group comparisons were conducted using a paired one-sided Bayesian Wilcoxon signed-rank test between intrusion and new trials (BF_Itemtype,0_ = 102)^7^ and a one-sided Bayesian *t* test (Cauchy prior with scale = .707) between intrusion and match trials (BF_Itemtype,0_ = .18). The proportion of trials with partial button presses was low (1.3% of valid trials; M partial_intrusion_ = 1.71%, SD = 2.54%; M partial_new_ = .16%, SD = .89%; M partial_match_ = 1.72%, SD = 1.95%). The results did not qualitatively change when we used the arcsine transformed data and the results were robust to changes in the width of the Cauchy prior. The results of the partial button presses should be interpreted cautiously as they are based on very few trials and the majority of participants did not show any partial errors in new and intrusion trials.

#### Non-preregistered analyses

The preregistered comparison of intrusion trials and new trials might not be the best comparison to assess the cost of response conflict. New trials can be resolved without using recollection altogether. Therefore, we also compared intrusion trials to match trials. Both require recollection but only the intrusion trials involve response conflict. We conducted a two (item type: intrusion vs. match; within-subjects) by two (validity of familiarity: low vs. high; between-subjects) Bayesian mixed effects ANOVA with JZS priors (Cauchy priors with scale = .5) on the participant mean RTs (Fig. 5A).

Comparing the main effects model (M_Main_) to the model with only the main effect of familiarity (M_Familiarity_) showed that the data is much more likely under M_Main_ (BF_Main,Familiarity_ = 2.1*10^5^). Therefore, we found strong evidence for intrusion costs in RTs (hypothesis 5; M RT_intrusion_ = 1216 ms, SD = 231 ms versus M RT_match_ = 1094 ms, SD = 200 ms). The comparison between the full model (M_Full_) and M_Main_ showed the data were slightly more likely under the model with vs without the familiarity × item type interaction (BF_Full,Main_ = 2.65) providing anecdotal evidence for the interaction. We did not further analyze partial button presses in the Modified Sternberg task due to their very rare occurrence.

## Discussion

Response conflict is an integral part of various psychological tasks. An established direct measure of response conflict is partial errors in the EMG. Here, we explored if analog keyboards could be used to assess partial errors. In line with the EMG findings of Seymour and Schumacher 2009, we picked up more partial errors in conflict trials than in control trials with the analog keyboards. Such partial errors were, however, rare. While we found the typical probe-irrelevant difference in RTs, we could not replicate the target familiarity effect (Suchotzki et al., 2018) despite having sufficient statistical power^8^. We therefore consider this response conflict manipulation unsuccessful. Similarly, we found intrusion costs in RTs and increased frequency of partial errors in the modified Sternberg task but ambiguous evidence regarding the response conflict manipulation.

While the response conflict manipulations would have helped to investigate the role of familiarity-based responding in more detail, we can still contrast conflict (probes; intrusions) to non-conflict (irrelevant; new, match) trials and compare the RT-CIT results to EMG findings.

### Comparison to EMG data

The comparison of our results to the EMG results of Seymour et al. (2009) shows qualitative similarities between keyboard and EMG partial errors (i.e., higher relative frequency of partial errors for probes than for irrelevant items) but also quantitative differences (probes: 28% EMG vs. 3% keyboard; irrelevants: 2% EMG vs. 0.5% keyboard). We see three possible reasons for this discrepancy.

First, and foremost, it seems likely that the analog keyboard is inherently less sensitive to detect partial errors than EMG. A factor possible contributing to the lower sensitivity is that we did not check whether participants adhered to the instruction to keep their fingers on the keyboard. This possibility could be addressed by filming the participant’s finger positions and excluding trials in which the fingers were not on the response keys, or by requiring that both response keys are minimally pressed for the next trial to start.

Second, there are a number of methodological differences between Seymour et al.’s EMG study and our analogue keyboard study. Most notable, we used deeply encoded autobiographical probes whereas Seymour and Schumacher (2009) used probes learned during the course of the study.

Third, based on the parallel task set model (Seymour, 2001), partial button presses would be expected to occur at a lower rate than EMG partial errors. According to this model, partial errors that can be detected by the analog keyboard occur only when response conflict is detected during the response execution step of the familiarity based response. The recording of sub-threshold muscular activity by the EMG, however, should also be sensitive to response conflict that is detected during the response preparation phase of the familiarity based response.

### Implications

For the RT-CIT, this method of detecting response conflict directly, especially the increased frequency of partial errors for probes compared to irrelevants, provides researchers with a new measure that could be used to detect knowledge in the RT-CIT. However, its incremental predictive value beyond RTs remains to be tested. Partial errors might also help detecting countermeasures such as intentionally slower responding (Norman et al., 2020; Suchotzki et al., 2021). We would expect that slower responding reduces the impact of familiarity and of the predominant ”no”-response which, in consequence, decreases the frequency of partial errors for both probes and targets.

On a more general note, the relatively large number of partial errors in target trials indicates that partial errors might have been significantly influenced by the tendency towards the predominant ”no”-response, given that five out of six trials required this response (e.g., Ratcliff & McKoon, 2008). It could be that this response bias made it more difficult to evoke familiarity-recollection-based partial errors. This suggests that the analog keyboard might be better suited for speeded conflict tasks with balanced responses (e.g., Eriksen flanker task, Simon task; Eriksen & Eriksen, 1974; Simon & Wolf, 1963).

The more detailed view on the response behavior provided by the analog keyboard and the occurrence of partial errors might call for extensions of contemporary response models. A widely used family of models, sequential sampling models (for a review, see Forstmann et al., 2016), generally assume that evidence accumulates over time until a decision threshold is reached upon which the motor response is initiated. These models successfully capture many characteristics of RT data but do not have mechanisms that could account for behavioral partial errors. Another model, the Parallel Task Set model (Seymour, 2001), predicts both pre-motor partial errors (e.g., measured with EMG) and behavioral partial errors due to conflicting response preparation of familiarity-based and recollection-based response. However, a discussion on how the models could be extended is out of the scope of this manuscript and would be premature given that the current study only provides a first glimpse at the pattern of partial errors.

### Future studies

This was the very first study to explore analog keyboards as an alternative to EMG to measure partial errors. Considering our results but also the quantitative difference to EMG partial errors (Seymour & Schumacher, 2009), follow-up studies should combine both measures to allow for a direct comparison and investigate if our results generalize to other speeded response conflict tasks (e.g., Erikson Flanker task, Simon task; Eriksen & Eriksen, 1974; Simon & Wolf, 1963).

We also urge researchers to independently replicate the target familiarity effect (Suchotzki et al., 2018) that has only been studied in two, although well-powered, online experiments (*n* = 357, *n* = 499) before and we failed to replicate. It would be valuable for researchers to know if this is a robust manipulation that can be used to manipulate the reliance on familiarity and therefore response conflict, and for practitioners have a way to improve the classification performance using familiar targets.

### Conclusions

Our study showed that analog keyboards can detect partial errors although they occurred in a small minority of conflict trials. Although likely less sensitive than EMG measures, potential benefits of analog keyboards include their accessibility, their compatibility with all tasks that use a standard keyboard, that no physical contact with the participant is needed, and ease of data collection (e.g., allowing for group testing). Analog keyboards could be a valuable tool to further our understanding of response conflict.

## Data Availability

The preregistration, material, data, and scripts that support the findings of this study are openly available on OSF (https://osf.io/x8ecn/).

## Footnotes

1 In the preregistration, they were called non-presented lures.

2 Items were adapted depending on the test site (German vs. Dutch cities and street names) and nationality (German vs. Dutch names).

3 To ensure that the BF_Main,Fam_ is not due to poor model fit of M_Fam_, we compared M_Main_ and M_Item_ to the null model (BF_Main,Null_ = 1.1*10^9^; BF_Item,Null_ = 1.4*10^9^).Therefore, M_Main_ and M_Item_ are considerably better fits to the data than the null model, confirming the effect of item type. This analysis was suggested by a reviewer and not preregistered.

4 To ensure that the BF_Main,Fam_ is not due to poor model fit of M_Fam_, we compared M_Main_ and M_Item_ to the null model (BF_Main,Null_ = 122; BF_Item,Null_ = 248).Therefore, M_Main_ and M_Item_ are considerably better fits to the data than the null model, confirming the effect of item type. This analysis was suggested by a reviewer and not preregistered.

5 To ensure that the BF_Main,Fam_ is not due to poor model fit of M_Fam_, we compared M_Main_ and M_Item_ to the null model (BF_Main,Null_ = 1.7*10^9^; BF_Item,Null_ = 1.4*10^9^).Therefore, M_Main_ and M_Item_ (and in extension M_Full_) are considerably better fits to the data than the null model. This analysis was not preregistered.

6 To ensure that the BF_Main,Fam_ is not due to poor model fit of M_Fam_, we compared M_Main_ and M_Item_ to the null model (BF_Main,Null_ = 40; BF_Item,Null_ = 121).Therefore, M_Main_ and M_Item_ are better fits to the data than the null model, confirming the effect of item type. This analysis was not preregistered.

7 Normality assumption of the preregistered *t* test was violated, we therefore report the results of the Wilcoxon signed rank test. Regardless, we also conducted the pairwise group comparison using paired one-sided Bayesian *t* test (Cauchy prior with scale = .707) between intrusion and new trials (BF_Itemtype,0_ = 115). The results do not differ qualitatively.

8 While we relied on Bayesian analysis for hypothesis testing, we conducted a frequentist power analysis to get a rough idea of the required sample size. Specifically, using G POWER 3.1. we calculated the power of a within-between interaction (post hoc) using the effect size of the two-way interaction between item and familiarity of Suchotzki et al.’s (2018) Experiment 1 (*np*^*2*^ = .19) , alpha = .05, *n* = 35, number of groups = 2, number of measurements = 2. This resulted in a power of > 99%. Experiment 2 of Suchotzki et al. (2018) found an effect size of (*np*^*2*^ = .25) and would, therefore, result in even higher power.

## References

[CR1] Allain S, Burle B, Hasbroucq T, Vidal F (2009). Sequential adjustments before and after partial errors. Psychonomic Bulletin and Review.

[CR2] Brainard, D. H. (1997). The psychophysics toolbox. *Spatial Vision, 10*, 433–436. 9176952

[CR3] Bürkner P-C (2017). brms : An R Package for Bayesian Multilevel Models Using Stan. Journal of Statistical Software.

[CR4] Coles, M. G. H., Gratton, G., Bashore, T. R., Eriksen, C. W., & Donchin, E. (1985). A psychophysiological investigation of the continuous flow model of human information processing. *Journal of Experimental Psychology: Human Perception and Performance, 11*(5), 529–553. 10.1037/0096-1523.11.5.529 10.1037//0096-1523.11.5.529 2932529

[CR5] Eriksen BA, Eriksen CW (1974). Effects of noise letters upon the identification of a target letter in a nonsearch task. Perception & Psychophysics.

[CR6] Eriksen, C. W., Coles, M. G. H., Morris, L. R., & O’hara, W. P. (1985). An electromyographic examination of response competition. *Bulletin of the Psychonomic Society, 23*(3), 165–168. 10.3758/BF03329816

[CR7] Farwell LA, Donchin E (1991). The Truth Will Out: Interrogative Polygraphy (“Lie Detection”) With Event-Related Brain Potentials. Psychophysiology.

[CR8] Forstmann BU, Ratcliff R, Wagenmakers EJ (2016). Sequential sampling models in cognitive neuroscience: Advantages, applications, and extensions. Annual Review of Psychology.

[CR9] Frank A, Biberci S, Verschuere B (2019). The language of lies: a preregistered direct replication of Suchotzki and Gamer (2018; Experiment 2). Cognition and Emotion.

[CR10] Gamer M, Verschuere B, Ben-Shakhar G, Meijer EH (2011). Detecting of deception and concealed information using neuroimaging techniques. *Memory detection: Theory and application of the Concealed Information Test*.

[CR11] Hadar AA, Makris S, Yarrow K (2012). The truth-telling motor cortex: Response competition in M1 discloses deceptive behaviour. Biological Psychology.

[CR12] Huster, R. J., Enriquez-Geppert, S., Lavallee, C. F., Falkenstein, M., & Herrmann, C. S. (2013). Electroencephalography of response inhibition tasks: Functional networks and cognitive contributions. *International Journal of Psychophysiology, 87*(3), 217–233. 10.1016/j.ijpsycho.2012.08.001 10.1016/j.ijpsycho.2012.08.001 22906815

[CR13] Koller, D., Hofer, F., & Verschuere, B. (2021). Different Target Modalities Improve the Single Probe Protocol of the Response Time-Based Concealed Information Test. *Journal of Applied Research in Memory and Cognition.*10.1016/j.jarmac.2021.08.003

[CR14] Langleben, D. D., Schroeder, L., Maldjian, J. A., Gur, R. C., McDonald, S., Ragland, J. D., ... Childress, A. R. (2002). Brain activity during simulated deception: An event-related functional magnetic resonance study. *NeuroImage, 15*(3), 727–732. 10.1006/nimg.2001.1003 10.1006/nimg.2001.1003 11848716

[CR15] Lemhöfer K, Broersma M (2012). Introducing LexTALE: A quick and valid Lexical Test for Advanced Learners of English. Behavior Research Methods.

[CR16] Li, Z. M., Latash, M. L., Newell, K. M., & Zatsiorsky, V. M. (1998). Motor redundancy during maximal voluntary contraction in four-finger tasks. *Experimental Brain Research, 122*(1), 71–78. 10.1007/s002210050492 10.1007/s002210050492 9772113

[CR17] Lukács, G., & Ansorge, U. (2021). The mechanism of filler items in the response time concealed information test. *Psychological Research, 85*(7), 2808–2828. 10.1007/s00426‐020‐01432‐y 10.1007/s00426-020-01432-y PMC8440312 33449206

[CR18] Lukács, G., & Steyrl, D. (2022). Machine learning mega-analysis applied to the response time concealed information test: No evidence for advantage of model-based predictors over baseline. *Collabra: Psychology, 8*(1), 1–12. 10.1525/collabra.32661

[CR19] Lukács G, Kleinberg B, Verschuere B (2017). Familiarity-Related Fillers Improve the Validity of Reaction Time-Based Memory Detection. Journal of Applied Research in Memory and Cognition.

[CR20] Lykken DT (1959). The GSR in the detection of guilt. Journal of Applied Psychology.

[CR21] MATLAB. (2018). *Version 9.4.0 (R2018a)*. The MathWorks Inc.

[CR22] Morey, R. D., & Rouder, J. N. (2018). *Computation of Bayes Factors for common designs (Version 4.2)* [Computer software]. https://richarddmorey.github.io/BayesFactor/. Accessed Jul 2022.

[CR23] Norman, D. G., Gunnell, D. A., Mrowiec, A. J., & Watson, D. G. (2020). Seen this scene? Scene recognition in the reaction-time Concealed Information Test. *Memory and Cognition.*10.3758/s13421-020-01063-z 10.3758/s13421-020-01063-z 32557195

[CR24] Nyberg, L., Marklund, P., Persson, J., Cabeza, R., Forkstam, C., Petersson, K. M., & Ingvar, M. (2003). Common prefrontal activations during working memory, episodic memory, and semantic memory. *Neuropsychologia, 41*(3), 371–377. 10.1016/S0028-3932(02)00168-9 10.1016/s0028-3932(02)00168-9 12457761

[CR25] Oberauer K (2001). Removing Irrelevant Information from Working Memory: A Cognitive Aging Study with the Modified Sternberg Task. Journal of Experimental Psychology: Learning Memory and Cognition.

[CR26] Olson JM, Rosenfeld PJ, Perrault E (2020). Familiarity-related filler items enhance the RT CIT (but not the P300 CIT) with differential effects on episodic compared to semantic protocols. International Journal of Psychophysiology.

[CR27] Quick Placement Test. (2001). Oxford University Press.

[CR28] R Core Team (2020). R: A language and environment for statistical computing. [Computer software]. https://www.R-project.org/

[CR29] Ratcliff R, McKoon G (2008). The Diffusion Decision Model: Theory and Data for Two-Choice Decision Tasks. Neural Computation.

[CR30] Rosenfeld JP, Cantwell G, Nasman VT, Wojdac V, Ivanov S, Mazzeri L (1988). A Modified, Event-Related Potential-Based Guilty Knowledge Test. International Journal of Neuroscience.

[CR31] Rosenfeld JP, Labkovsky E, Winograd M, Lui MA, Vandenboom C, Chedid E (2008). The Complex Trial Protocol (CTP): A new, countermeasure-resistant, accurate, P300-based method for detection of concealed information. Psychophysiology.

[CR32] Sai, L., Cheng, J., Shang, S., Fu, G., Verschuere , B. (2022). Does Deception involves more cognitive control? A meta-analyses of ERP studies. Working paper. 10.1111/psyp.14333 37194343

[CR33] Schumacher EH, Seymour TL, Schwarb H (2010). Brain activation evidence for response conflict in the exclude recognition task. Brain Research.

[CR34] Seymour TL (2001). A EPIC model of the “guilty knowledge effect”: *Strategic and automatic processes in recognition. Dissertation Abstracts International: Section B*. The Sciences & Engineering.

[CR35] Seymour TL, Schumacher EH (2009). Electromyographic evidence for response conflict in the exclude recognition task. Cognitive, Affective and Behavioral Neuroscience.

[CR36] Seymour TL, Seifert CM, Shafto MG, Mosmann AL (2000). Using response time measures to assess “guilty knowledge”. Journal of Applied Psychology.

[CR37] Simon J, Wolf JD (1963). Choice Reaction Time As A Function Of Angular Stimulus-Response Correspondence And Age. Ergonomics.

[CR38] Strange BA, Henson RNA, Friston KJ, Dolan RJ (2000). Brain mechanisms for detecting perceptual, semantic, and emotional deviance. NeuroImage.

[CR39] Suchotzki K, De Houwer J, Kleinberg B, Verschuere B (2018). Using more different and more familiar targets improves the detection of concealed information. Acta Psychologica.

[CR40] Suchotzki, K., Verschuere, B., & Gamer, M. (2021). How Vulnerable is the Reaction Time Concealed Information Test to Faking? *Journal of Applied Research in Memory and Cognition, January.*10.1016/j.jarmac.2020.10.003

[CR41] Suchotzki K, Verschuere B, Peth J, Crombez G, Gamer M (2015). Manipulating item proportion and deception reveals crucial dissociation between behavioral, autonomic, and neural indices of concealed information. Human Brain Mapping.

[CR42] Verschuere B, Kleinberg B (2016). ID-Check: Online Concealed Information Test Reveals True Identity. Journal of Forensic Sciences.

[CR43] Verschuere B, Meijer EH (2014). What’s on your mind? Recent advances in memory detection using the concealed information test. European Psychologist.

[CR44] Visu-Petra G, Miclea M, Buş I, Visu-Petra L (2014). Detecting concealed information: The role of individual differences in executive functions and social desirability. Psychology, Crime & Law.

[CR45] Visu-Petra G, Miclea M, Visu-Petra L (2012). Reaction time-based detection of concealed information in relation to individual differences in executive functioning. Applied Cognitive Psychology.

[CR46] Yonelinas AP (2002). The nature of recollection and familiarity: A review of 30 years of research. Journal of Memory and Language.

[CR47] Zhang R, Geng X, Lee TMC (2017). Large-scale functional neural network correlates of response inhibition: an fMRI meta-analysis. Brain Structure and Function.

